# Survival and deterioration time of walking abilities in dogs homozygous for the SOD1 gene mutation with and without thoracolumbar intervertebral disc protrusion

**DOI:** 10.3389/fvets.2025.1555889

**Published:** 2025-05-09

**Authors:** Péter Sebestyén, Malwina Ewa Kowalska, Lorenzo Golini

**Affiliations:** ^1^Department of Small Animals, Section of Neurology, Vetsuisse Faculty Zurich, University of Zurich, Zurich, Switzerland; ^2^Equine Department, Section of Ophthalmology, Vetsuisse Faculty Zurich, University of Zurich, Zurich, Switzerland; ^3^Section of Epidemiology, Vetsuisse Faculty Zurich, University of Zurich, Zurich, Switzerland

**Keywords:** canine, magnetic resonance imaging, spinal cord disease, degenerative myelopathy, Kaplan–Meier survival analysis

## Abstract

**Introduction:**

Dogs homozygous for the SOD1 gene mutation with presumptive degenerative myelopathy (DM) can develop concurrent intervertebral disc protrusion (IVDP). The impact of IVDP on the progression of SOD1-related clinical signs is unknown. The aim of this study was to describe a population of dogs with the SOD1 mutation and to compare survival and time to non-ambulation between those with and without IVDP.

**Methods:**

This single-center exploratory cohort study was preregistered and retrospectively included dogs with the SOD1 gene mutation, compatible clinical signs, and available spinal magnetic resonance imaging (MRI). Dogs were divided into two groups based on the presence (IVDP+) or absence (IVDP-) of IVDP affecting the T3-L3 spinal cord segment. The primary outcomes were time to euthanasia from the onset of clinical signs (neurological deficits) and from the diagnosis (genetic testing and MRI). The secondary outcome was time to non-ambulatory status. Data were analyzed using descriptive statistics and survival analysis.

**Results:**

A total of 39 dogs were enrolled in the study, with a mean age of 115 months and a mean weight of 29 kg at the time of diagnosis. The most common breed was the German Shepherd (*n* = 9/39). In the IVDP- group (*n* = 28/39), the median survival time was 13 months (95% CI: 9–18 months) from the onset of clinical signs, and 6 months (95% CI: 5–11 months) from the time of diagnosis. In the IVDP+ group (*n* = 11/39), the median survival time was 11 months (95% CI: 9-∞ months) from the onset of clinical signs, and 7 months (95% CI: 5-∞ months) from the diagnosis. Cox regression analysis indicated that dogs with IVDP had a hazard ratio of 1.20 for euthanasia (95% CI: 0.58–2.49, *p* = 0.6), which was not statistically significant compared to dogs without IVDP.

**Discussion:**

Based on this retrospective cohort, dogs with the SOD1 mutation appear to have similar disease progression and survival, regardless of the presence of concurrent IVDP.

**Clinical trial registration:**

The study has been preregistered on https://preclinicaltrials.eu/ (PCT ID: PCTE0000406).

## 1 Introduction

Superoxide dismutase 1 (SOD1) is an intracellular antioxidant enzyme (1). Mutations in the SOD1 gene that cause misfolding of the protein’s quaternary structure have been linked to neurodegeneration (2). Homozygosity for the glutamate-to-lysine transition in exon 2 (c. 118G > A) of the SOD1 gene in most dog breeds and homozygosity for the threonine-to-serine transition in exon 1 (c. 52A > T) of the same gene in the Bernese Mountain Dog have been identified as a major risk factor for the development of degenerative myelopathy (DM) (3–5).

Degenerative myelopathy is a progressive and fatal neurodegenerative disease of the spinal cord in middle-aged and older dogs (6–9). Neurological deficits usually appear after 5 years of age, but in large breed dogs the median age of onset is approximately 9 years (7, 10). The clinical signs initially consist of general proprioceptive ataxia and spastic paraparesis without spinal hyperesthesia. These signs progress to paraplegia, then to flaccid tetraplegia, and ultimately to respiratory compromise if euthanasia is delayed (3, 6, 7, 11). Dogs may become non-ambulatory between 6 and 12 months and may develop bulbar signs and sudden death 48 months after the first clinical signs appear (3, 10).

Antemortem diagnosis is presumptive and based on signalment, clinical signs and further diagnostic investigations such as medical imaging, cerebrospinal fluid analysis and electrodiagnostic, to rule out other etiologies (12). Identification of the mutated SOD1 gene can also support clinical diagnosis. However, DM is an incompletely penetrant autosomal recessive disease and not every dog with homozygosity for the SOD1 mutation develops clinical signs (3). For a definitive diagnosis, a histopathological examination of the spinal cord is necessary (6, 7, 9). No medical therapy has been shown to be beneficial in the treatment of DM, however physiotherapy may delay its progression (13–15).

Dogs affected by DM can develop concurrent intervertebral disc protrusion (IVDP) (13). This pathology is characterized by slowly progressive chondroid metaplasia of the nucleus pulposus (NP) and degeneration of the annulus fibrosus (AF), resulting in focal extension of the NP and AF into the spinal canal and compression of the spinal cord (16–19). Thoracolumbar IVDP is also common in older, non-chondrodystrophic large breed dogs and can result in clinical signs similar to those seen in the early stages of DM (17, 18). To the authors’ best knowledge, the impact of this comorbidity on survival and speed of deterioration in walking abilities in dogs with presumptive DM has not been investigated (13). This information would be crucial because shorter survival and a shorter time to non-ambulatory status in dogs with the SOD1 mutation and concurrent IVDP might impact clinical decision making and potential treatment recommendations.

This study aimed to describe a population of dogs with the SOD1 mutation and to compare survival (time to euthanasia) and speed of deterioration of walking abilities (time to non-ambulatory status) between those with and without a concurrent IVDP.

## 2 Materials and methods

### 2.1 Study population and inclusion criteria

This single-center, retrospective cohort study included dogs presented to the Vetsuisse Faculty of the University of Zurich, Switzerland, between December 1, 2012, and October 31, 2024. To identify eligible cases, electronic health records (EHR) were screened for the SOD1 genetic testing. Dogs were included if they had complete medical records (including at least the variables listed below) at the time of presentation to the referral hospital, had clinical signs consistent with progressive thoracolumbar (T3-L3) myelopathy, and were homozygous for a transition in exon 2 (c. 118G > A) and/or exon 1 (c. 52A > T) of the SOD1 gene, had available magnetic resonance imaging scan of at least the thoracolumbar (T3-L3) spinal cord, as well as contact information of caregivers and referring veterinarians. Dogs euthanized for reasons unrelated to DM or IVDP (e.g., hemoabdomen due to neoplasia of the spleen) or comorbidities that may have affected ambulation (e.g., severe orthopedic disease) were excluded.

Due to the retrospective nature of the study, ethical approval was not required. Written consent was obtained from the caregiver for the use of the animal’s data in veterinary clinical research.

### 2.2 Clinical, laboratory and imaging data

Variables extracted from EHR included: breed; sex; neutering status; onset of clinical signs; age, body weight, clinical signs including spinal hyperesthesia, and neuroanatomical localization at the time of genetic testing and MRI; medical history including details of other chronic diseases as well as the type, duration and outcome of therapy initiated prior to diagnosis.

Neurological signs indicative of thoracolumbar (T3-L3) myelopathy include general proprioceptive ataxia and upper motor neuron paraparesis. These manifestations are characterized by varying degrees of motor dysfunction in the hindlimbs, ranging from ambulatory to non-ambulatory paraparesis. Additional clinical features may include postural reaction deficits affecting one or both hindlimbs, normal or exaggerated spinal reflexes in the hindlimbs, increased or normal muscle tone, and, in some cases, hyperesthesia (20). The severity of neurological signs at the time of diagnosis and at the time of euthanasia was graded according to the modified Frankel Score (MFS) as follows: 5 for paraplegics with absent nociception, 4 for paraplegics with nociception, 3 for non-ambulatory paraparetics, 2 for ambulatory paraparetics, 1 for spinal pain only, and 0 for no neurological deficits (21, 22). Non-ambulatory status was defined as the inability to walk at least 10 steps without assistance (23). Clinical and neurological examination was performed by a board-certified neurologist or a neurology resident under the direct supervision of a board-certified neurologist.

A commercial laboratory (LABOKLIN, Germany) conducted genetic testing on EDTA samples using the polymerase chain reaction (PCR) method for either exon 2 (c. 118G > A) or both exon 1 (c. 52A > T) and exon 2 (c. 118G > A) of SOD1 according to the breed.

Magnetic resonance imaging of spinal cord was performed under general anesthesia using a 3-Tesla scanner (Philips Ingenia, Philips AG, Zurich, Switzerland). The images were reviewed by a board-certified radiologist and the imaging protocol consisted of a minimum of T2W sequence in the sagittal and transverse planes and one T2W-SPIR sequence in the dorsal plane. Cases were included when an IVDP was present, based on established MRI criteria, and when there was at least moderate spinal cord compression (i.e., compressive material occupying more than 25% of the cross section of the spinal canal) between the T3 and L3 spinal cord segment (24–26). Dogs were divided into two groups based on the presence (IVDP+) or absence (IVDP-) of disc protrusion(s). The number and location of IVDP was also recorded.

### 2.3 Follow-up information

Follow-up data were collected from EHR and structured telephone interviews with either the referring veterinarians or both the caregivers and the referring veterinarians responsible for the cases. Questions were about the type, frequency, and duration of physiotherapy with a qualified animal physiotherapist following diagnosis; any additional treatment performed; timing of non-ambulatory status; neurological grade at euthanasia; and the reason and date on which euthanasia was carried out.

### 2.4 Study outcomes

The primary outcomes were the time in months from onset of clinical signs and from diagnosis to euthanasia. The secondary outcomes were the time in months from onset of clinical signs and from diagnosis to non-ambulatory status. The onset of clinical signs was defined as the time when neurological deficits (e.g., proprioceptive ataxia and impaired ambulation) first appeared, and diagnosis was defined as the time when both MRI and genetic test results were first available.

### 2.5 Statistical analysis

Descriptive analysis was performed to examine possible differences between the IVDP+ and IVDP- groups. This was supported by calculating a 95% confidence interval (95% CI). The distribution of continuous variables was assessed using histograms. When normally distributed, they were reported as median, mean, standard deviation (SD), and range (minimum to maximum). Categorical variables were presented as proportions.

The authors chose to use a 95% CI in the descriptive analysis to increase the generalizability of our results. Variables for which the 95% CI ranges between IVDP+ and IVDP- dogs do not overlap are factors that may be statistically significant in the larger, adequately powered dog population and therefore may influence survival.

Survival analysis was conducted to determine the median time from onset of clinical signs and diagnosis to euthanasia and non-ambulatory status, supported by Kaplan–Meier plots stratified by IVDP status. Animals were censored if they were lost to follow-up or remained ambulatory at the time of euthanasia (for the secondary outcome). The log-rank test was used to assess whether the difference in survival between the two groups (IVDP+ and IVDP-) was statistically significant. Univariate and multivariate Cox proportional hazard analysis were performed to examine the influence of the variables (spinal hyperesthesia, physiotherapy and IVDP) on the survival. The analysis was carried out in the R statistical software (version 4.0.5, www.R-project.org) using the DescTools, readxl, dplyr, ggplot2, gtsummary, and survminer packages (27–32).

The authors aimed to gather initial evidence and investigate the relationship between IVDP and survival, as well as IVDP and deterioration time of walking abilities in dogs homozygous for the SOD1 mutation. Therefore, the study is descriptive and exploratory in nature. For this type of research, the sample size is not required. In contrast to studies using hypothesis testing (confirmatory research), where a predefined number of enrolled cases (i.e., sample size) is required to ensure adequate power. In exploratory research, the use of hypothesis testing is incorrect and results in biased results, that can ultimately mislead the reader. Therefore, the authors would like to emphasize that we used Cox proportional hazard analysis in an exploratory manner. The reported *p*-value should be interpreted with caution for the reason mentioned above. Causal relationships between the outcome of interest and variables in Cox analysis cannot be definitively confirmed or rejected.

## 3 Results

### 3.1 Study population

A total of 149 genetic tests were performed during the study period (December 1, 2012 to October 31, 2024). Dogs were excluded due to the absence of SOD1 homozygosity (*n* = 70/149), lack of an MRI scan (*n* = 36/149), IVDP with moderate compression affecting the L4-S3 spinal cord segment (*n* = 1/149) or severe comorbidities (*n* = 3/149). All dogs underwent SOD1 genetic testing and MRI scanning at the referral hospital.

Thirty-nine cases met all inclusion criteria. The study population comprised 54% females (*n* = 21/39; 16 spayed and 5 sexually intact) and 46% males (*n* = 18/39; 13 neutered and 5 sexually intact). The most common breed was the German Shepherd (*n* = 9; 23%), followed by mixed breed (*n* = 6; 15.5%), Rough Collie (*n* = 3; 7.7%), and equal number of Fox Terrier, Boxer, Saarloos Wolfhound, Hovawart and Soft Coated Wheaten Terrier (each *n* = 2; 5.1%). Details of the other breeds can be found in Supplementary Table S1.

**Figure 1:**
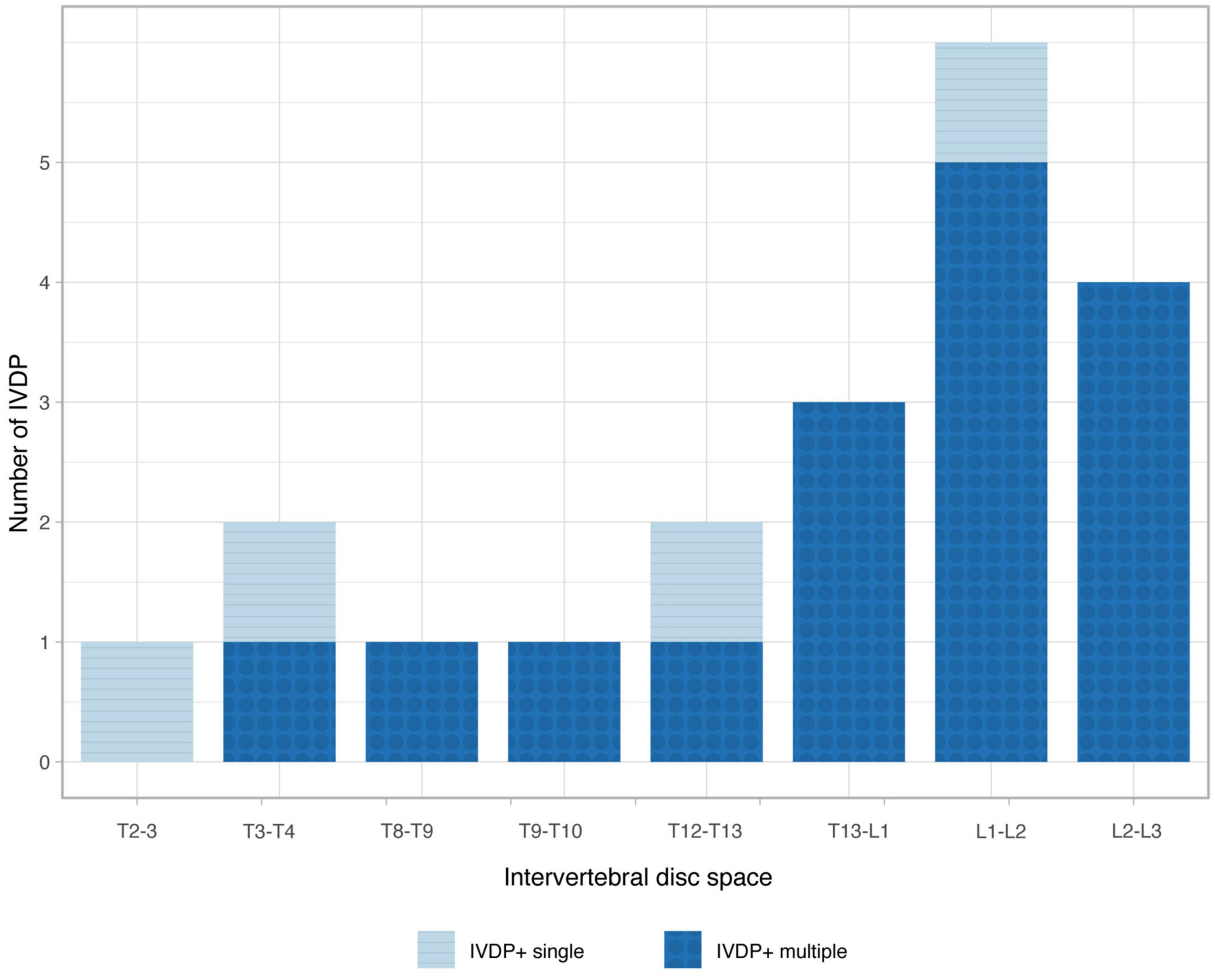
Bar chart illustrating the frequency of intervertebral disc protrusion (IVDP) at various locations in the single-site (light blue) and multiple-site (dark blue) IVDP+ groups.

Based on the MRI findings, the population was divided into IVDP+ (*n* = 11/39; 28%) and IVDP- (*n* = 28/39; 72%) groups. In the IVDP+ group, 4/11 (36%) dogs had a single, and 7/11 (64%) dogs had multiple thoracolumbar IVDP. Cases depending on the number and location of IVDP are summarized in Figure 1. No other causes of compressive myelopathy (e.g., subarachnoid diverticulum) were identified in the included cases.

### 3.2 Clinical data at diagnosis

In the IVDP- group, the mean age at diagnosis was 116 months (approximately 9.5 years; SD 22, range: 80–142 months) and the mean weight was 27 kg (SD 9, range: 12–48 kg). Dogs in this group showed clinical signs before the diagnosis for a mean of 4.6 months (SD 3.3, range: 1–12 months) and 64% (*n* = 18/28) of them received medical treatment, consisting of nonsteroidal anti-inflammatory drugs (NSAID) (*n* = 14/28; 50%), gabapentin (*n* = 5/28; 18%), prednisolone (*n* = 2/28; 7%), pregabalin (*n* = 2/28; 7%), bedinvetmab (*n* = 2/28; 7%), paracetamol (*n* = 1/28; 3.5%), as well as acupuncture (*n* = 2/28; 7%) and physiotherapy (*n* = 1/28; 3.5%) alone or as part of a multimodal therapy. The mean duration of treatment before diagnosis was 2.7 months (SD 2.89, range: 0–4) and only one dog showed short-term improvement with prednisolone. Twenty-six dogs (92%) had a neurological grade compatible with MFS 2, while 2/28 dogs (8%) already had MFS 3 at the time of diagnosis. Neurological signs were lateralized in 64% of cases (*n* = 18/28; Table 1).

**Table 1:** Descriptive statistical summary of demographic information, clinical signs and treatment until diagnosis in dogs with SOD1 gene mutation.

		**IVDP-** (*n* = 28)	**IVDP+** (*n* = 11)	**Overall** (*n* = 39)
***Signalments***				
**Age at diagnosis [months] (mean ± SD)**		116 ± 22	114 ± 21	115 ± 21
**Bodyweight at diagnosis [kg] (mean ± SD)**		27 ± 9	33 ± 10	29 ± 10
**Sex**	Intact females	3 (11%)	2 (18%)	5 (13%)
	Spayed females	11 (39%)	5 (45%)	16 (41%)
	Intact males	3 (11%)	2 (18%)	5 (13%)
	Neutered males	11 (39%)	2 (18%)	13 (33%)
***At presentation to the referral hospital***				
**Duration of clinical signs until the diagnosis [months] (mean ± SD)**		4.6 ± 3.3	6.5 ± 4.9	5.2 ± 3.8
**Clinical signs (MFS) at diagnosis**	MFS 2	26 (93%)	10 (91%)	36 (92%)
	MFS 3	2 (7%)	1 (9%)	3 (8%)
	Others	0 (0%)	0 (0%)	0 (0%)
**Spinal hyperesthesia**	Yes	9 (32%)	6 (55%)	15 (38%)
	No	19 (68%)	5 (45%)	24 (62%)
**Neurological deficits**	Lateralized	18 (64%)	7 (64%)	25 (64%)
	Symmetrical	10 (36%)	4 (36%)	14 (36%)
**Neurological deficits compatible with MRI findings**	Ipsilateral	N/A	3 (27%)	N/A
	Contralateral	N/A	8 (73%)	N/A
***Treatment until the diagnosis***				
**Treatment type**	Medical	18 (64%)	6 (55%)	24 (62%)
	Surgical	0 (0%)	1 (9%)	1 (3%)
	None	10 (34%)	4 (36%)	14 (35%)
**Duration of the medical treatment [weeks] (mean ± SD)**		2.71 ± 2.89	1.64 (1.75)	2.41 ± 2.64
**Short-term improvement with medical treatment other than prednisolone**	Yes	0 (0%)	2 (18%)	2 (5%)
	No	10 (36%)	4 (36%)	14 (36%)
	Not treated	18 (64%)	5 (45%)	23 (59%)
**Short-term improvement with prednisolone**	Yes	1 (3%)	0 (0%)	1 (3%)
	No	3 (11%)	2 (18%)	5 (13%)
	Not used	24 (86%)	9 (82%)	33 (85%)

Abbreviations: IVDP-, intervertebral disc protrusion absent; IVDP+, intervertebral disc protrusion present; MFS, modified Frankel Score; MRI, magnetic resonance imaging; N/A, not applicable; SD, standard deviation; SOD1, Superoxide dismutase 1.

In the IVDP+ group, the age at diagnosis was similar with a mean of 114 months (approximately 9.5 years; SD 21, range: 82–150 months). These dogs had a mean weight of 33 kg (SD 10, range: 7–48 kg) and exhibited clinical signs for a longer period, with a mean of 6.5 months (SD 4.9, range: 1–17 months). Most of them (*n* = 6/11; 55%) received medical therapy, being treated with NSAID (*n* = 5/11; 45%), prednisolone (*n* = 2/11; 18%) gabapentin (*n* = 2/11; 18%), and amantadine (*n* = 1/11; 9%), as well as acupuncture (*n* = 1/11; 9%). This group received medications for a shorter duration, with a mean treatment period of 1.64 months (SD 1.75, range: 0–5 months). Only two dogs showed short-term improvement with NSAID. One dog underwent decompression surgery before the SOD1 status was known, but no improvement was observed thereafter. At diagnosis, MFS 2 was the most common neurological grade (*n* = 10/11; 91%), and spinal hyperesthesia was also a frequently reported clinical finding (*n* = 6/11; 55%). Neurological signs were lateralized in 64% (*n* = 7/11), with 42% of cases (*n* = 3/7) consistent with the side of the IVDP identified on MRI (Table 1).

### 3.3 Follow-up information

Follow-up data was available for 35/39 (90%) of cases. Four cases from the IVDP- group (*n* = 4/28; 14%) were lost to follow-up after MRI and genetic testing were performed and data could only be extracted up to the time of diagnosis. The outcome was determined based on a re-examination at the referral hospital for 5/35 dogs (14%) and a follow-up discussion with referring veterinarians and caregivers for 30/35 dogs (86%). All dogs with available follow-up had been euthanized at the time of data collection. No postmortem histopathological examination was available for the included cases.

In the IVDP- group, 50% of the dogs (*n* = 12/24) received physiotherapy from a qualified animal physiotherapist, mostly every other week (*n* = 5/12; 21%) for a mean duration of 3.3 months (SD 4.9, range: 0–16 months). Additionally, 7 dogs (28%) in this group underwent underwater treadmill therapy. In the IVDP+ group a slightly higher percentage, with 55% of the dogs (*n* = 6/11), received physiotherapy, usually on a weekly basis (*n* = 3/11; 27%), along with underwater treadmill (*n* = 4/11; 36%). However, the duration of physiotherapy in this group was shorter with a mean of 2.0 months (SD 2.2, range: 0–6 months). Further details on physiotherapy can be found in Supplementary Table S2.

In the IVDP- group, the most common neurological grade at euthanasia was MFS 4, reported in 46% of dogs (*n* = 11/24), followed by MFS 3, observed in 33% of dogs (*n* = 8/24). However, 13% of dogs (*n* = 3/24) were still at MFS 2 when euthanasia was performed at the caregiver’s request. In the IVDP+ group, MFS 3 and MFS 4 were the most common grades (each *n* = 5/11; 45%) at the time of euthanasia, although 1/11 dog (10%) was still at MFS 2.

In the IVDP- group, the most common cause of euthanasia (*n* = 20/24; 84%) was the general loss of quality of life as perceived by the caregivers, with some (*n* = 2/24; 8%) also citing fecal incontinence as a reason. In the IVDP+ group, caregivers primary chose euthanasia due to a general loss of quality of life (*n* = 7/11; 64%), with urinary incontinence being a terminating factor for 18% (*n* = 2/11) of them (Supplementary Table S3).

### 3.4 Survival analysis

**Figure 2:**
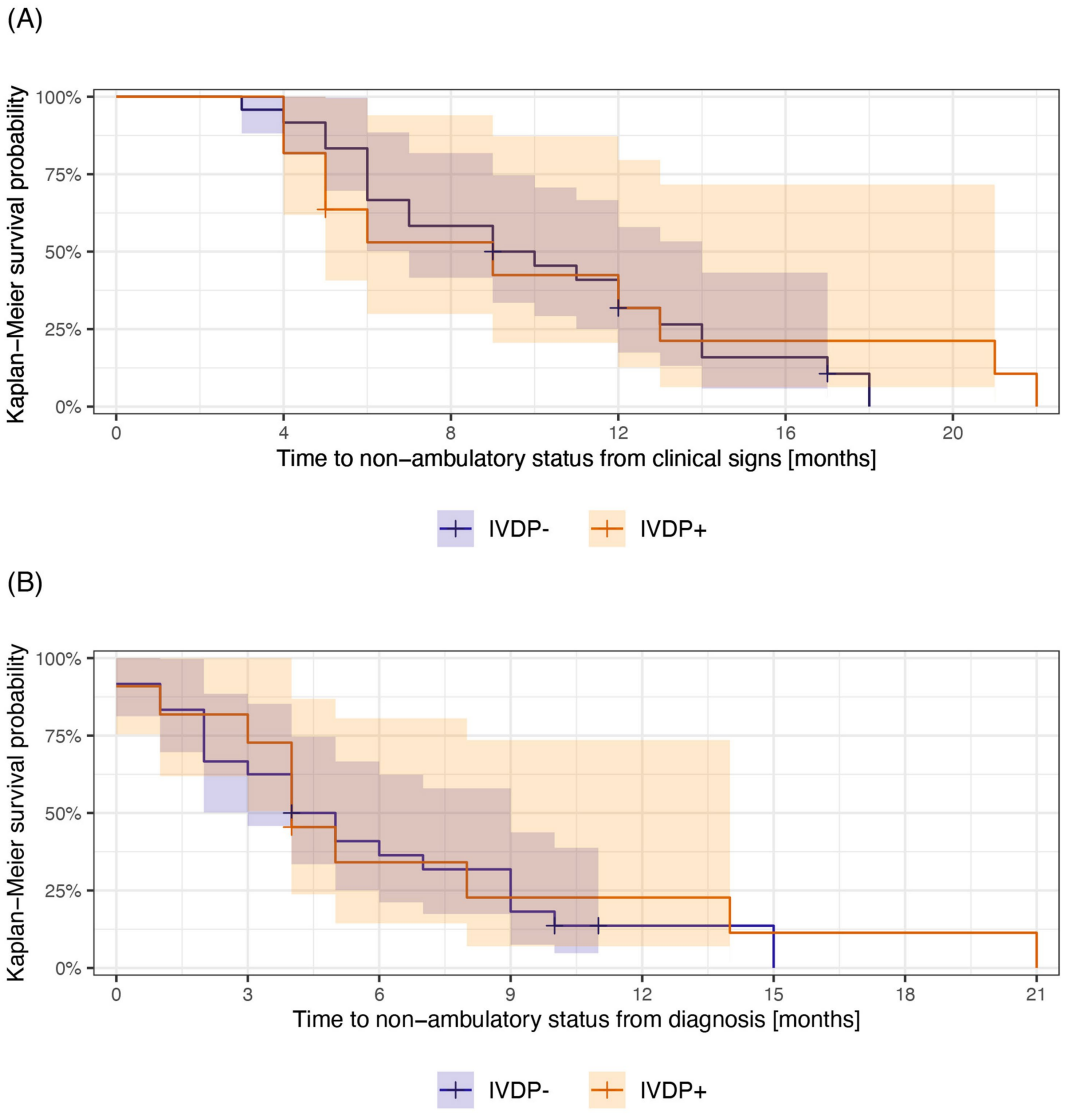
Kaplan–Meier survival curves illustrating survival from the onset of clinical signs **(A)** and diagnosis **(B)** to euthanasia in the IDVP- (blue) and IVDP+ (orange) groups. The median survival time for each group is the length of time corresponding to 50% survival probability. Shadow areas indicate 95% CI around the survival probabilities at a given time. The “+” indicates the time at which a case was censored.

The median survival time for the entire study population was 12 months (95% CI: 9–16 months) from the onset of clinical signs and 7 months (95% CI: 5–10 months) from the diagnosis (Table 2). In the IVDP- group, the median survival time was 13 months (95% CI: 9–18 months) from the onset of clinical signs and 6 months (95% CI: 5–11 months) from diagnosis. In the IVDP+ group, the median survival time was similar: 11 months (95% CI: 9-∞ months) from the clinical signs and 7 months (95% CI: 5-∞ months) from diagnosis (Figure 2).

**Table 2:** Primary and secondary outcomes.

	**IVDP-** (*n* = 28)	**IVDP+** (*n* = 11)	**Overall** (*n* = 39)
***Primary outcome***			
**Median time to euthanasia from onset of clinical signs (95% CI)**	13 (9-18)	11 (9-∞)	12 (9-16)
**Median time to euthanasia from diagnosis (95% CI)**	6 (5-11)	7 (5-∞)	7 (5-10)
***Secondary outcome***			
**Median time to non-ambulatory status from onset of clinical signs (95% CI)**	9.5 (7-14)	9 (5-∞)	9 (6-13)
**Median time to non-ambulatory status from diagnosis (95% CI)**	4.5 (3-9)	4 (4-∞)	4 (4-8)

Abbreviations: IVDP-, intervertebral disc protrusion absent; IVDP+, intervertebral disc protrusion present; 95% CI, 95% confidence interval.

**Figure 3:**
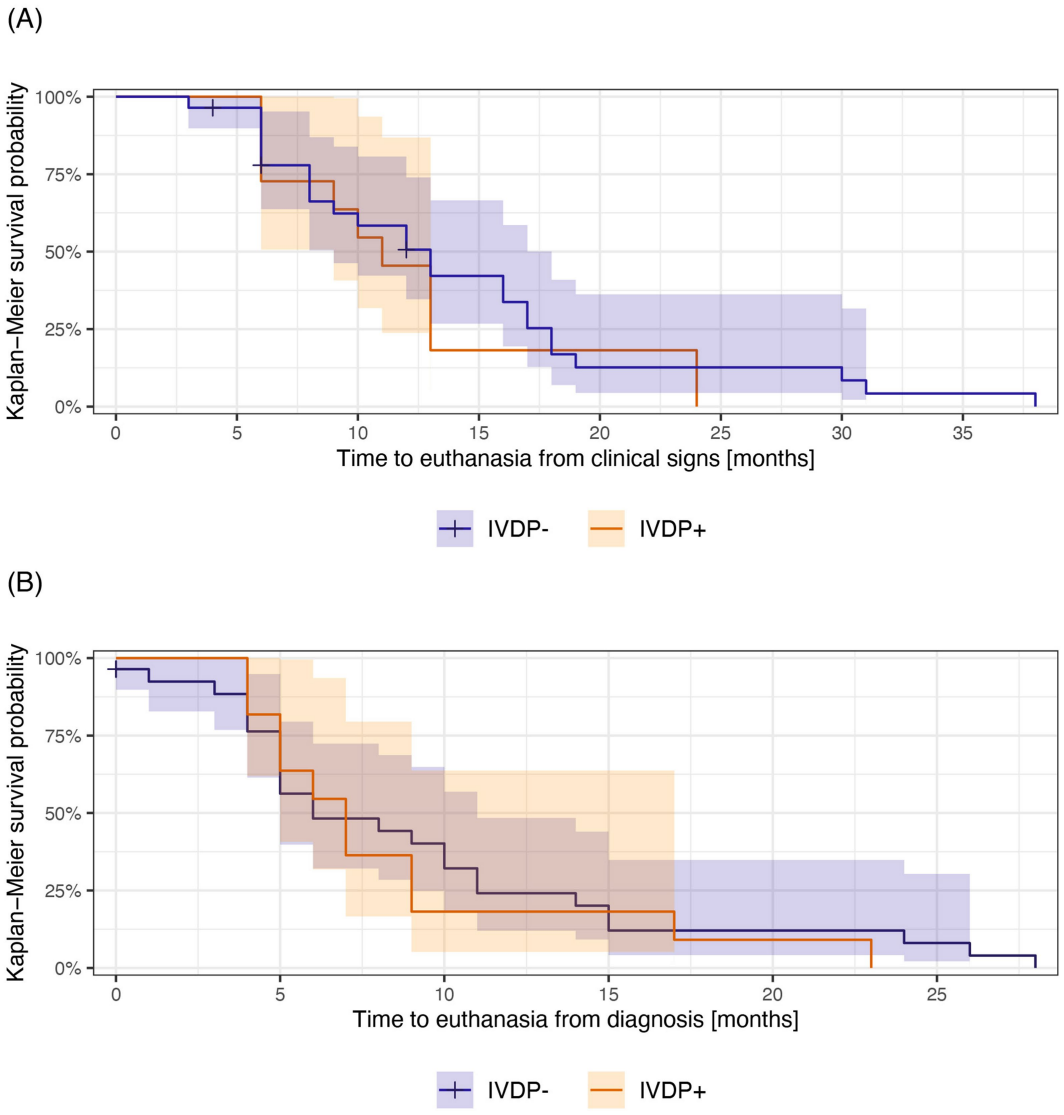
Kaplan–Meier survival curves illustrating survival from the onset of clinical signs **(A)** and diagnosis **(B)** to non-ambulatory status in IVDP- (blue) and IDVP+ (orange) groups. The median time to non-ambulatory status for each group is the length of time corresponding to 50% survival probability. Shadow areas indicate 95% CI around survival probabilities at a given time. The “+” indicates the time at which a case was censored.

The median time to non-ambulatory status for the study population was 9 months (95% CI: 6–13 months) from the onset of clinical signs and 4 months (95% CI: 4–8 months) from the diagnosis (Table 2). The IVDP- group had a median time to becoming non-ambulatory of 9.5 months (95% CI: 7–14 months) from clinical signs and 4.5 months (95% CI: 3–9 months) from the diagnosis. Similarly, the IVDP+ group had a median time to non-ambulation of 9 months (95% CI: 5-∞ months) from onset of clinical signs and 4 months (95% CI: 4-∞ months) from the diagnosis (Figure 3). The upper limit of the confidence intervals could not be estimated due to the small number of cases.

There was no statistically significant difference between the two groups, with a *p*-value of 0.6. The Kaplan–Meier curves showed a large overlap for primary (Figure 2) and secondary outcomes (Figure 3) based on both clinical signs (Figure 2A and Figure 3A) and diagnosis (Figure 2B and Figure 3B), with no apparent divergence in survival time and probabilities over time.

Both univariate and multivariate Cox proportional hazard analysis revealed no significant association between survival time and variables such as spinal hyperesthesia (yes/no), physiotherapy (yes/no) and IVDP status (IVDP+/IVDP-) (Table 3). The univariate hazard ratio (HR) for IVDP status comparing the IVDP+ and IVDP- groups was 1.20 (95% CI: 0.58–2.49) with a *p*-value of 0.6. This suggests that the risk of euthanasia may be higher in the IVDP+ group, although not statistically significant.

**Table 3:** Results of the univariate and multivariate Cox regression analysis.

	**Hazard ratio (HR)**	***p-*value**
***Univariate analysis***		
**IVDP**	1.20 (0.58-2.49)	0.6
**Physiotherapy**	1.12 (0.57-2.21)	0.7
**Spinal hyperesthesia**	0.90 (0.45-1.81)	0.8
***Multivariate analysis***		
**IVDP + Physiotherapy**	1.09 (0.55-2.17)	0.8
**IVDP + Spinal hyperesthesia**	0.83 (0.39-1.75)	0.6

Abbreviations: HR, hazard ratio; IVDP, intervertebral disc protrusion; 95% CI, 95% confidence interval.

## 4 Discussion

This study, to the authors’ best knowledge, is the first to investigate the impact of IVDP on survival and time to non-ambulation in dogs with the SOD1 mutation. A total of 28% of dogs in this cohort had a concurrent IVDP. The statistical analysis did not find sufficient evidence to indicate that the presence of IVDP significantly shortens survival time or accelerates the progression to non-ambulatory status compared to dogs without IVDP. Similarly, neither spinal hyperesthesia nor the practice of physiotherapy appeared to have a statistically meaningful influence on survival time.

The signalment, clinical presentation and time to non-ambulatory status of the included dogs in both groups were similar to those reported previously (6, 7, 10, 16, 33). The study population consisted mainly of larger, older, non-chondrodystrophic dogs, with the German Shepherd being the most affected by presumptive DM (n = 9/39; 23%) and concurrent IVDP (n = 6/9; 66%). Most dogs were presented with progressive ambulatory paraparesis and proprioceptive ataxia and reached non-ambulatory status in median time of 9 months (95% CI: 6–13 months) from onset of clinical signs.

Spinal hyperesthesia was observed in 55% of the cases in the IVDP+, suggesting that the presence of IVDP, associated with focal pain due to spinal cord compression, may still be clinically significant in dogs with presumptive DM, which typically presents as non-painful myelopathy (6, 7). However, based on our statistical analysis this was not associated with a shorter survival (HR 0.83; 95% CI: 0.39–1.75; *p* = 0.6). Lateralization of clinical signs was also common in both groups. In the IVDP+ group, neurological deficits were consistent with the side of compression in 42% of the cases, further supporting the potential clinical relevance of IVDP in this context.

Degenerative myelopathy has long been considered a natural animal model for Lou Gehrig’s disease, or genetic amyotrophic lateral sclerosis (ALS), due to the SOD1 mutation and its accumulation in motor neurons, similar to what is observed in humans (34). From this perspective, it is interesting to note that the dilemma of concurrent disc disease in patients with ALS in human medicine mirrors the dilemma of DM with concurrent IVDP in veterinary medicine. Both diseases can cause similar clinical signs and occur simultaneously in the same patients, requiring a clinical decision on further treatment. The prevalence of concurrent intervertebral disc disease is also high in humans, occurring in up to 48% of ALS patients (35). Studies in human medicine suggest that surgical treatment of intervertebral disc disease may slow the progression of ALS-related disease in some cases but may also accelerate it in others (35–37).

Most of the enrolled cases received medical treatment before genetic testing and MRI were performed, but in the majority, this did not lead to improvement in clinical signs. According to the literature, there is no proven medical therapy that reduces the progression of clinical signs in dogs with degenerative myelopathy (13, 14). In this cohort, 2/11 dogs from the IVDP+ group showed short-term improvement. We hypothesize that in these cases, analgesics relieved the pain associated with spinal cord compression during the acute phase and resulted in clinical improvement. Nevertheless, the long-term progression of neurological deficits was likely driven by the underlying pathology of DM.

There is no consensus on the treatment of IVDP in dogs. Due to its chronic nature and the frequent occurrence of compression at multiple sites, surgical treatment of the disease can be challenging and may be associated with neurological deterioration in the postoperative period (38). A retrospective study comparing medically and surgically treated dogs with thoracolumbar IVDP reported a higher long-term success rate in surgically treated dogs (38). In our study population, only one dog was treated surgically for IVDP, but in this case, there was no improvement in clinical signs, and the dog was euthanized shortly after genetic testing results were available.

The positive influence of physiotherapy on prolonging survival time in dogs with suspected DM was reported in a retrospective study, although cases involving concurrent IVDP were not included (15). In our population, most dogs also received physiotherapy after the presumptive diagnosis of DM. However, this factor did not show a significant effect in the survival analysis (HR 1.09; 95% CI: 0.55–2.17; *p* = 0.8), likely also due to the small sample size.

Our study had several limitations, including a small sample size, a retrospective data without a predefined standardized data collection sheet, unbalanced groups, and cases lost to follow-up. Recall bias regarding the time between onset of clinical signs and the time of euthanasia, as well as potential misclassification between MFS grades by referring veterinarians were also possible.

Furthermore, DM is an age-dependent, incompletely penetrant autosomal recessive disease. Homozygosity for the SOD1 gene is a major risk factor for developing the disease and may support *in vivo* diagnosis. However, not all dogs homozygous for the mutant allele will exhibit clinical signs or develop the disease, and a definitive diagnosis can only be made postmortem. Given the presumptive DM diagnosis and the lack of histopathological examination, DM may also have been misdiagnosed in this cohort.

The quality of life of the dogs, which played a pivotal role in euthanasia decisions, was subjectively assessed by their caregivers. While some caregivers were willing to take care for a dog using mobility aids, others considered the loss of walking ability a decisive factor for euthanasia. The authors suggest that this subjective perception of quality of life and the caregiver burden may influence the survival time of affected dogs and should be explored further in future studies.

In conclusion, our findings suggests that IVDP might be a common occurrence in dogs with presumptive DM. Based on this cohort, dogs with the SOD1 mutation and concurrent IVDP appeared to have slightly higher risk of euthanasia; however, this difference was not statistically significant. Similarly, the time to non-ambulatory status was also comparable regardless of the presence of IVDP. Nevertheless, cases with spinal hyperesthesia and clinical signs compatible with MRI findings indicate that IVDP can still be a clinically relevant condition. Therefore, medical treatment should be considered to improve the quality of life of these dogs, at least in the short-term.

Prospective, multicenter studies with larger sample sizes and balanced groups, employing standardized treatment approaches for IVDP are necessary to better understand and establish firm conclusions regarding survival outcomes, time to non-ambulatory status and the potential benefit of additional treatment (e.g., physiotherapy, medical management, or surgery) in dogs with the SOD1 mutation and concurrent IVDP.

## Data Availability

The collected data and statistical analysis are available in the online repository on Open Science Framework at https://osf.io/dymw9/?view_only=691c9b7846044cc8b6ea5f9aa685b28b.
