# Supplementary material for: Survival and deterioration time of walking abilities in dogs homozygous for the SOD1 gene mutation with and without thoracolumbar intervertebral disc protrusion
Source: Front Vet Sci. 2025 May 9;12:1555889. doi: 10.3389/fvets.2025.1555889 (PMC12098292; doi:10.3389/fvets.2025.1555889)
Supplement: Supplementary file 1 [file Table_1.docx]

Supplementary Material

# Supplementary Tables

**Supplementary Table 1.** Dog breed distribution in the study population

|  | **IVDP-**, n = 28 | **IVDP+**, n = 11 | **Overall**, n = 39 |
| --- | --- | --- | --- |
| ***Breeds*** | | | |
| **German shepherd dog** | 3 (11%) | 6 (55%) | 9 (23%) |
| **Mixed breed dog** | 6 (21%) | 0 (0%) | 6 (15.5%) |
| **Rough Collie** | 3 (11%) | 0 (0%) | 3 (7.7%) |
| **Fox Terrier** | 1 (3.5%) | 1 (9%) | 2 (5.1%) |
| **Saarloos Wolfdog** | 1 (3.5%) | 1 (9%) | 2 (5.1%) |
| **Boxer** | 2 (7.5%) | 0 (0%) | 2 (5.1%) |
| **Hovawart** | 1 (3.5%) | 1 (9%) | 2 (5.1%) |
| **Soft Coated Wheaten Terrier** | 2 (7.5%) | 0 (0%) | 2 (5.1%) |
| **Tibetian Terrier** | 1 (3.5%) | 0 (0%) | 1 (2.5%) |
| **Siberian Husky** | 1 (3.5%) | 0 (0%) | 1 (2.5%) |
| **Bernese Mountain Dog** | 0 (0%) | 1 (9%) | 1 (2.5%) |
| **Epagneul Français** | 1 (3.5%) | 0 (0%) | 1 (2.5%) |
| **Podenco Ibicenco** | 1 (3.5%) | 0 (0%) | 1 (2.5%) |
| **Andalusian Hound** | 1 (3.5%) | 0 (0%) | 1 (2.5%) |
| **White Swiss Shepherd Dog** | 0 (0%) | 1 (9%) | 1 (2.5%) |
| **Komondor** | 1 (3.5%) | 0 (0%) | 1 (2.5%) |
| **Pembroke Welsh Corgi** | 1 (3.5%) | 0 (0%) | 1 (2.5%) |
| **French Bulldog** | 1 (3.5%) | 0 (0%) | 1 (2.5%) |
| **Czechoslovakian Wolfdog** | 1 (3.5%) | 0 (0%) | 1 (2.5%) |
| Abbreviations: IVDP-, intervertebral disc protrusion absent; IVDP+ intervertebral disc protrusion present | | | |

**Supplementary Table 2.** Physiotherapy after diagnosis in dogs with available follow-up

|  | **IVDP-**, n = 24 | **IVDP+**, n = 11 | **Overall**, n = 35 |
| --- | --- | --- | --- |
| ***Physiotherapy*** | | | |
| **Performed**  Yes  No | 12 (50%)  12 (50%) | 6 (55%)  5 (45%) | 18 (51%)  17 (49%) |
| **Frequency**  Once a week  Twice a week  Every other week  Not performed | 4 (17%)  3 (13%)  5 (20%)  12 (60%) | 3 (27%)  2 (18%)  1 (9%)  5 (45%) | 7 (20%)  5 (14%)  6 (17%)  17 (49%) |
| **Underwater treadmill**  Yes  No  Unknown | 7 (29%)  16 (67%)  1 (4%) | 4 (36%)  7 (64%)  0 (0%) | 11 (31%)  23 (66%)  1 (3%) |
| **Length of the treatment [months] (mean ± SD)** | 3.3 ± 4.9 | 2.0 ± 2.2 | 2.9 ± 4.3 |
| Abbreviations: IVDP-, intervertebral disc protrusion absent; IVDP+, intervertebral disc protrusion present; SD, standard deviation | | | |

**Supplementary Table 3.** Clinical signs at euthanasia and cause of euthanasia in dogs with available follow-up

|  | **IVDP-**, n = 24 | **IVDP+**, n = 11 | **Overall**, n = 35 |
| --- | --- | --- | --- |
| ***Clinical signs (MFS) at euthanasia*** | | | |
| **MFS 2**  **MFS 3**  **MFS 4**  **MFS 5** | 3 (13%)  8 (33%)  11 (46%)  2 (8%) | 1 (10%)  5 (45%)  5 (45%)  0 (0%) | 4 (11%)  13 (37%)  16 (46%)  2 (6%) |
| ***Cause of euthanasia*** | | | |
| **Progressive neurological deficits: general loss of quality of life perceived by the caregivers**  **Progressive neurological deficits: urinary incontinence**  **Progressive neurological deficits: urinary and fecal incontinence**  **Complications related to neurological deficits: maggot infestation.**  **Acute neurological deterioration after acupuncture** | 20 (84%)  2 (8%)  1 (4%)  1 (4%)  0 (0%) | 7 (64%)  2 (18%)  0 (0%)  1 (9%)  1 (9%) | 27 (77%)  4 (11%)  1 (3%)  2 (6%)  1 (3%) |
| Abbreviations: IVDP-, intervertebral disc protrusion absent; IVDP+, intervertebral disc protrusion present; MFS, modified Frankel score | | | |
